# RETRACTION: Effect of Chitosan‐Based Gel Dressing on Wound Infection, Synechia, and Granulations after Endoscopic Sinus Surgery of Nasal Polyps: A Meta‐Analysis

**DOI:** 10.1111/iwj.70424

**Published:** 2025-03-26

## Abstract

**RETRACTION:** LiuR. and GongZ. , “Effect of Chitosan‐Based Gel Dressing on Wound Infection, Synechia, and Granulations after Endoscopic Sinus Surgery of Nasal Polyps: A Meta‐Analysis,” International Wound Journal 19, no. 8 (2022): 2146–2153, 10.1111/iwj.13820.35524493 PMC9705184

The above article, published online on 07 May 2022, in Wiley Online Library (http://onlinelibrary.wiley.com/), has been retracted by agreement between the journal Editor in Chief, Professor Keith Harding; and John Wiley & Sons Ltd. Following an investigation by the publisher, all parties have concluded that this article was accepted solely on the basis of a compromised peer review process. The editors have therefore decided to retract the article. The authors did not respond to our notice regarding the retraction.

**RETRACTION:** R. Liu and Z. Gong , “Effect of Chitosan‐Based Gel Dressing on Wound Infection, Synechia, and Granulations after Endoscopic Sinus Surgery of Nasal Polyps: A Meta‐Analysis,” International Wound Journal 19, no. 8 (2022): 2146–2153, 10.1111/iwj.13820.35524493 PMC9705184

The above article, published online on 07 May 2022, in Wiley Online Library (http://onlinelibrary.wiley.com/), has been retracted by agreement between the journal Editor in Chief, Professor Keith Harding; and John Wiley & Sons Ltd. Following an investigation by the publisher, all parties have concluded that this article was accepted solely on the basis of a compromised peer review process. The editors have therefore decided to retract the article. The authors did not respond to our notice regarding the retraction.

